# Diagnostic value of symptoms and signs for identifying urinary tract infection in older adult outpatients: Systematic review and meta-analysis

**DOI:** 10.1016/j.jinf.2018.06.012

**Published:** 2018-11

**Authors:** Oghenekome A. Gbinigie, José M. Ordóñez-Mena, Thomas R. Fanshawe, Annette Plüddemann, Carl Heneghan

**Affiliations:** Nuffield Department of Primary Care Health Sciences, University of Oxford, Radcliffe Primary Care Building, Radcliffe Observatory Quarter, Woodstock Road, Oxford OX2 6GG, United Kingdom

**Keywords:** Urinary tract infection, Older adults, Diagnosis, Symptoms and signs

## Abstract

**Objectives:**

To critically appraise and evaluate the diagnostic value of symptoms and signs in identifying UTI in older adult outpatients, using evidence from observational studies.

**Methods:**

We searched Medline and Medline in process, Embase and Web of Science, from inception up to September 2017. We included studies assessing the diagnostic accuracy of symptoms and/or signs in predicting UTI in outpatients aged 65 years and above. Study quality was assessed using the QUADAS-2 tool.

**Results:**

We identified 15 eligible studies of variable quality, with a total of 12,039 participants (range 65–4259), and assessed the diagnostic accuracy of 66 different symptoms and signs in predicting UTI. A number of symptoms and signs typically associated with UTI, such as nocturia, urgency and abnormal vital signs, were of limited use in older adult outpatients. Inability to perform a number of acts of daily living were predictors of UTI: For example, disability in feeding oneself, + ve LR: 11.8 (95% CI 5.51–25.2) and disability in washing one's hands and face, + ve LR: 6.84 (95% CI 4.08–11.5).

**Conclusions:**

The limited evidence of varying quality shows that a number of symptoms and signs traditionally associated with UTI may have limited diagnostic value in older adult outpatients.

## Highlights

• Older adults may present atypically with UTI and making a diagnosis can be difficult.
• There is limited authoritative guidance on how older adult outpatients present with UTI.
• Symptoms and signs traditionally associated with UTI (e.g. nocturia, urgency and abnormal vital signs) may have limited utility in diagnosing these infections in older adult outpatients.
• Disability in performing a number of acts of daily living may be better predictors of UTI; high quality studies should be conducted in this area to confirm this.

## Background

Life expectancy is increasing and the population of older people is growing: people over 65 years constitute one sixth of the population, but account for one in three outpatient attendances.^1^ Moreover, the older population is at increased risk of bacterial infections, which can cause significant morbidity, mortality and further exacerbate hospital attendance. Of the bacterial infections, urinary tract infection (UTI) is the commonest in older adults.^2^ If not treated promptly, sepsis may ensue. Each year in the UK there are 150,000 cases of sepsis in the population, causing 44,000 deaths.^3^ UTI is the commonest cause of sepsis in older adults,^4^ with urinary sepsis causing an in-hospital mortality of 33% in this age group.^2^ In the United States (US), sepsis is the tenth leading cause of death in patients over 65.^5^

UTI is the leading cause of emergency hospital admissions for acute conditions that could effectively be managed in the community.^6^ Over 65 s account for three times as many admissions to hospital for UTI compared to younger adults.6 The cost of hospitalizations is significant; in the UK, hospitalisations for UTI cost £316 million per annum respectively.^7^ Adults in the US aged 65–84 years account for over a quarter of hospital stays, and compared to other age groups have the highest average cost per stay at $12,300.^8^ Preventing hospital admission for UTI requires early recognition and treatment. However, UTI in older adults often presents atypically, which can lead to diagnostic uncertainty. A study of older adults in primary care found that UTI was the second most common infection initially missed by clinicians,^9^ with many of the reasons for not making the diagnosis at the earliest possible opportunity related to problems with history taking. Symptoms and signs typically associated with infection in younger patients, such as fever, might be absent in older people.^10^ Of note, however, asymptomatic bacteriuria is common in older adults and should not be treated with antimicrobials.^11^

Currently, there is a lack of authoritative guidance to aid clinicians in making accurate diagnoses when UTI presents atypically. For example, there is no specific NICE guidance relating to the clinical features of UTI in older adults. The Scottish Intercollegiate Guidelines Network (SIGN) recognises that typical symptoms used to diagnose UTI, such as dysuria and urinary frequency, may not apply to all frail, older women in whom atypical presentations are common.^12^ However, these atypical presentations are not fully explained within the guideline. In order to effectively diagnose and treat UTI in older adult outpatients, a clearer understanding of the clinical features that predict infections is required. The aim of this systematic review is therefore to determine the clinical features associated with UTI in older adult outpatients.

## Methods

### Search strategy

We performed searches in Medline and Medline in process, Embase and Web of Science, from inception up to September 2017 (See supplementary file 1 for full strategy). We also conducted searches of the bibliography of retrieved full texts. Two reviewers (OAG and JMOM) independently determined study eligibility. Disagreements were resolved through discussion, and where controversy remained, the opinion of a third reviewer was sought (TRF).

### Inclusion criteria

We included studies: of cohort and cross-sectional design; assessing the diagnostic accuracy of symptoms and/or signs in predicting UTI; providing a reference standard for confirming diagnosis of infection; conducted in the outpatient setting of developed countries (e.g. General Practice, nursing homes, and outpatient clinics); and in patients over 65 years. We included studies in which a small proportion of participants were aged under 65 years. If the age range of participants was not clear from the full text, we contacted the authors of studies to clarify.

To meet our inclusion criteria studies had to provide data to enable construction of two by two tables for calculation of diagnostic accuracy parameters (e.g. sensitivity and specificity).

### Exclusion criteria

We excluded studies: of immunosuppressed participants (such as active cancer or receiving chemotherapy); conducted in developing countries, as we anticipated considerable variation in the timing and mode of presentation of UTI, such that findings may not be applicable to developed countries; not published in English; with non-human subjects; and systematic reviews, case reports, case series and conference abstracts. Systematic reviews were used as a point of reference. We excluded studies conducted in Accident and Emergency (A&E) Units. Whilst A&E units forms part of outpatient care, the prevalence of serious disease in A&E is likely to be higher than in settings outside of hospital.

### Quality assessment

Two reviewers (OAG and JMOM) independently assessed the quality of included studies according to the Quality Assessment of Diagnostic Accuracy Studies-2 (QUADAS-2) tool.^13^ Disagreements were resolved through discussion, and where controversy remained, the opinion of a third assessor was sought (AP).

### Data extraction and analysis

Two reviewers (OAG and JMOM) independently extracted data from individual studies into two by two tables. When it was not possible from the available information to extract data for individual participants, episodic data was used; one participant may have experienced more than one illness episode. Any discrepancies were resolved through discussion, or the opinion of a third reviewer was sought (TRF). We calculated sensitivity, specificity, positive and negative likelihood ratios, and pre- and post-test probabilities of disease for each symptom or sign in relation to UTI. When an empty cell rendered these calculations impossible, a continuity correction factor of 0.5 was added in the table.

Where the diagnostic accuracy of a clinical feature in predicting UTI could be assessed across four or more studies, we plotted the results in receiver operating characteristic (ROC) space^14^ and, unless heterogeneity were very high, estimated pooled sensitivity and specificity and summary ROC curves were produced using the bivariate model^15^ in STATA v.13.1 (StataCorp, College Station, TX) with the “metandi” command.^16^ We estimated heterogeneity between studies through visual inspection of the ROC plots, assessment of the 95% prediction region around the summary operating point, and the 95% confidence region. When more than one definition of the same symptom or sign was reported by the same study, we used the definition that was most similar to those described by other studies for pooling results. We planned to perform subgroup and sensitivity analyses to further investigate significant heterogeneity, however, we did not have sufficient included studies to allow for meaningful evaluation.

We present all other results narratively and on a dumbbell plot derived in Microsoft Excel (Redmond, WA).^14^ The plots include the pre-test probability (UTI prevalence), and the post-test probability of UTI given presence or absence of the symptom or sign in question (derived using the positive or the negative likelihood ratio, respectively). Study specific estimates were sorted into categories based on the type of symptom or sign reported and separated according to gender, if gender-specific results were available. We classed symptoms as helpful rule in or rule out tests for UTI if the LR + or LR- were statistically significant, respectively.

## Results

**Fig. 1:**
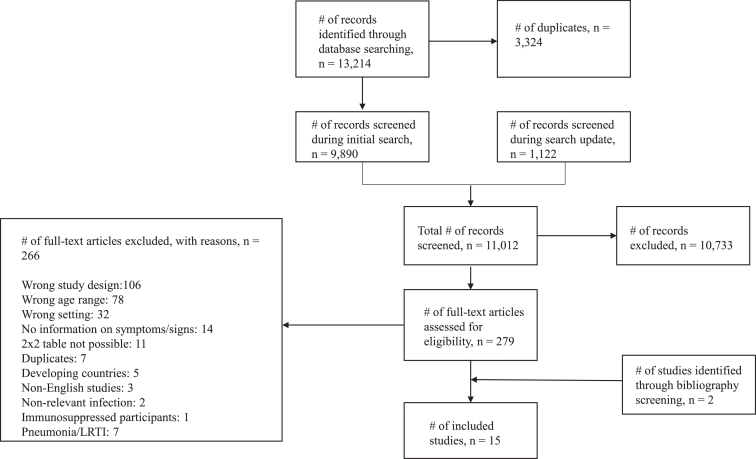
Flow chart showing the process for identification of studies eligible for inclusion.

Our initial search in February 2016 identified 9890 non-duplicate results. The search was updated in September 2017, and an additional 1122 non-duplicate results were identified. 279 eligible studies were identified through title and abstract screening, and after full text screening we included 15 studies (Fig. 1)**.** As we wished to investigate clinical predictors of a number of different bacterial infections, in order to economize the article screening process, we included in our search strategy all bacterial infections that were of interest to us (see search strategy, supplementary file 1). We therefore excluded seven studies that focused on pneumonia/lower respiratory tract infection (LRTI), and have not included them in the analyses reported here.^17^, ^18^, ^19^, ^20^, ^21^, ^22^, ^23^

Details of the 15 studies reported in this review are shown in Table 1. The studies included 12,039 participants (range 65–4259 per study). Four were cohort studies^24^, ^25^, ^26^, ^27^ and the rest were cross-sectional.^28^, ^29^, ^30^, ^31^, ^32^, ^33^, ^34^, ^35^, ^36^, ^37^, ^38^ Nine studies were conducted in Europe^24^, ^28^, ^29^, ^30^, ^31^, ^32^, ^33^, ^36^, ^37^ and six in North America.^25^, ^26^, ^27^, ^34^, ^35^, ^38^ Locations included nursing homes (six studies), residential care facilities (one study), long-term care facilities (one study), participants’ homes (three studies) and combinations of the aforementioned settings (four studies). In four studies, a small number of participants had cancer.^24^, ^25^, ^26^, ^30^

**Table 1:** Characteristics of included studies. CFU: colony-forming units; N/A: not applicable; UTI: urinary tract infection; WBC: white blood cell count.

Author, year and country	Study type	Study setting	Number of participants	Age (years)	Study duration	Reference test
Bjornsdottir et al. (1998) Iceland^28^	Cross-sectional	Home/nursing homes	110	80–89	N/A	Positive urine cultures, history of antibiotic treatment for UTI and recorded UTI in notes
Brocklehurst et al. (1968) England^29^	Cross-sectional	Home setting	557	≥65	N/A	Bacteriuria (CFU > 100,000/ml)
Caljouw et al. (2011) The Netherlands^24^	Cohort	Home/long term care facility	479	86	4 years	Physician diagnosis of UTI based on signs, symptoms, urine analysis, and death from UTI
Carlsson et al. (2013) Sweden^30^	Cross-sectional	Residential care facilities	188	65–100	N/A	Documented symptomatic UTI with antibiotic treatment. UTI diagnosis supported by previous lab tests or bacterial cultures
Daley et al. (2015) Canada^25^	Cohort	Long-term care facility	101	≥65	3 months	Urine culture: > 10^8 CFU/L of uropathogenic bacteria
Eriksson et al. (2011) Sweden and Finland^31^	Cross-sectional	Homes/institutions	504	≥85	N/A	Combination of diagnosis of UTI in notes, and suggestive symptoms/lab results
Eriksson et al. (2010) Sweden and Finland^32^	Cross-sectional	Homes/institutions	395	≥85	N/A	Documented UTI diagnosis in the medical records from the GP/hospital and records from the caring institutions
Heudorf et al. (2012) Germany^33^	Cross-sectional	Nursing homes	3732	11% under 65 years	N/A	Adapted McGeer criteria, thus physician diagnosis of infection was included as a criterion in all categories of infection to avoid under-estimation of the infection rate due to lack of on-site diagnostic testing. Only 17 of the 39 UTI cases had tests; 14 had a dipstick, 3 had a culture
Juthani-Mehta et al. (2009) USA^26^	Cohort	Nursing homes	551	> 65	2 years	Urine culture (Defined as bacteriuria of > 100,000 CFU plus pyuria defined as > 10 WBCs) combined with urinalysis
Lara et al. (1990) USA^27^	Cohort	Nursing home care unit	99	Unclear	N/A	Bacteriuria (over 100,000 bacterial colony count/ml) - clean-catch of catheterized urine specimens
Magaziner et al. (1991) USA^34^	Cross-sectional	Long term care facilities/ Nursing homes	4259	>65	N/A	A combination of symptoms/signs/lab investigations. Not all patients had a urine culture
Midthun et al. (2004) USA^35^	Cross-sectional	Nursing homes	97	64–102	N/A	Two different definitions used: Bacteriuria alone (≥ 50,000 CFU/ml growth of a single organism) or Bacteriuria and Pyuria (> 10 WBCs/hpf)
Sourander et al. (1965) Finland^36^	Cross-sectional	Recruited from home setting, examinations performed in outpatient department of the Municipal Hospital of Turku	481	≥65	N/A	Growth > 10^5 bacteria/ml in clean voided urine
Sundvall et al. (2014) Sweden^37^	Cross-sectional	Nursing homes	421	63–100	N/A	Urine culture of ≥ 10^5 CFU/ml OR ≥ 10^3 if E.coli growth or in male patients with Klebsiella/enterococcus faecalis OR ≥ 10^4 in women growing Klebsiella/enterococcus faecalis
Whippo et al. (1989) USA^38^	Cross-sectional	Nursing homes	65	64–97	N/A	Urine culture > 100,000 bacteria/ml urine

### Risk of bias

**Fig. 2:**
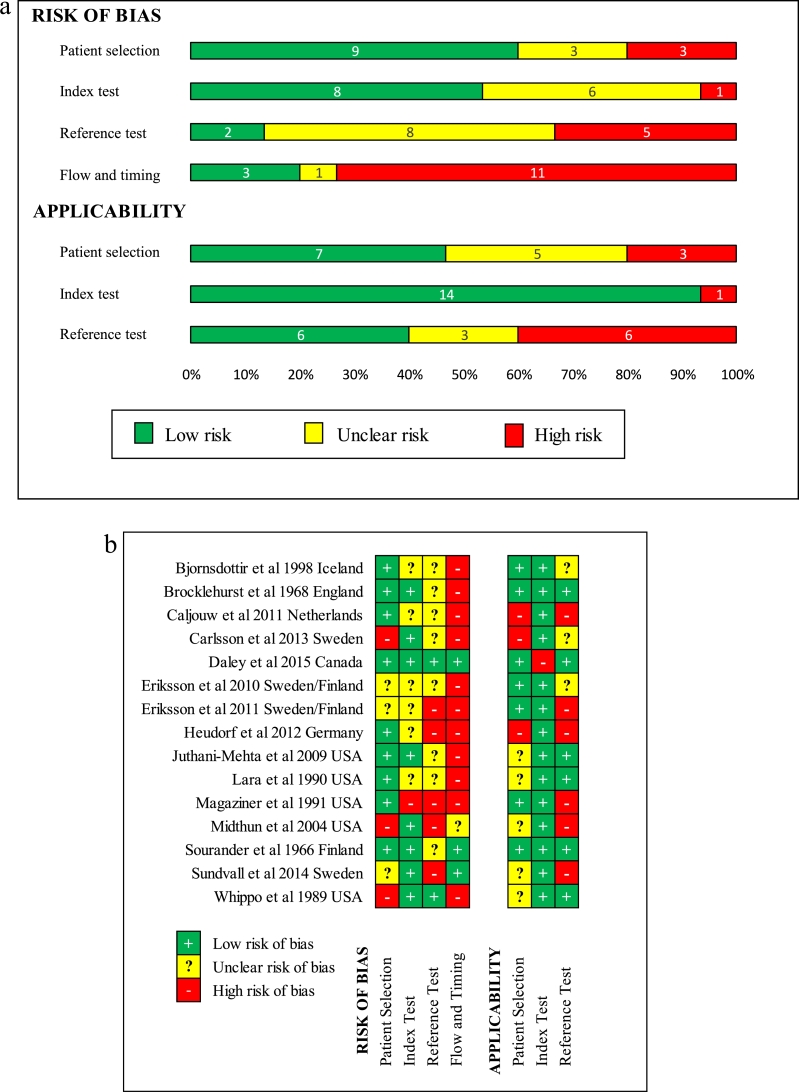
(a) Risk of bias graph. (b) Risk of bias summary.

Fig. 2a and 2b show that none of the included studies were assessed as low risk of bias against all of the domains in the QUADAS-2 tool.^13^ Three studies (20%) were assessed as low risk of bias with regard to the flow and timing of the index and reference tests^25^, ^36^, ^37^ and nine had low risk of bias with regard to patient selection.^24^, ^25^, ^26^, ^27^, ^28^, ^29^, ^33^, ^34^, ^36^ Two were low risk with regard to the reference test;^25^, ^38^ for many studies it was unclear whether the reference standard was interpreted with knowledge of the index test results (symptoms and signs). Applicability of the index test to the review question was assessed as unclear or high risk of bias in only one study.^25^ Six studies were rated as high risk due to prolonged intervals between index test and reference standard.^24^, ^28^, ^30^, ^31^, ^32^, ^33^

### Symptoms and signs

**Fig. 3:**
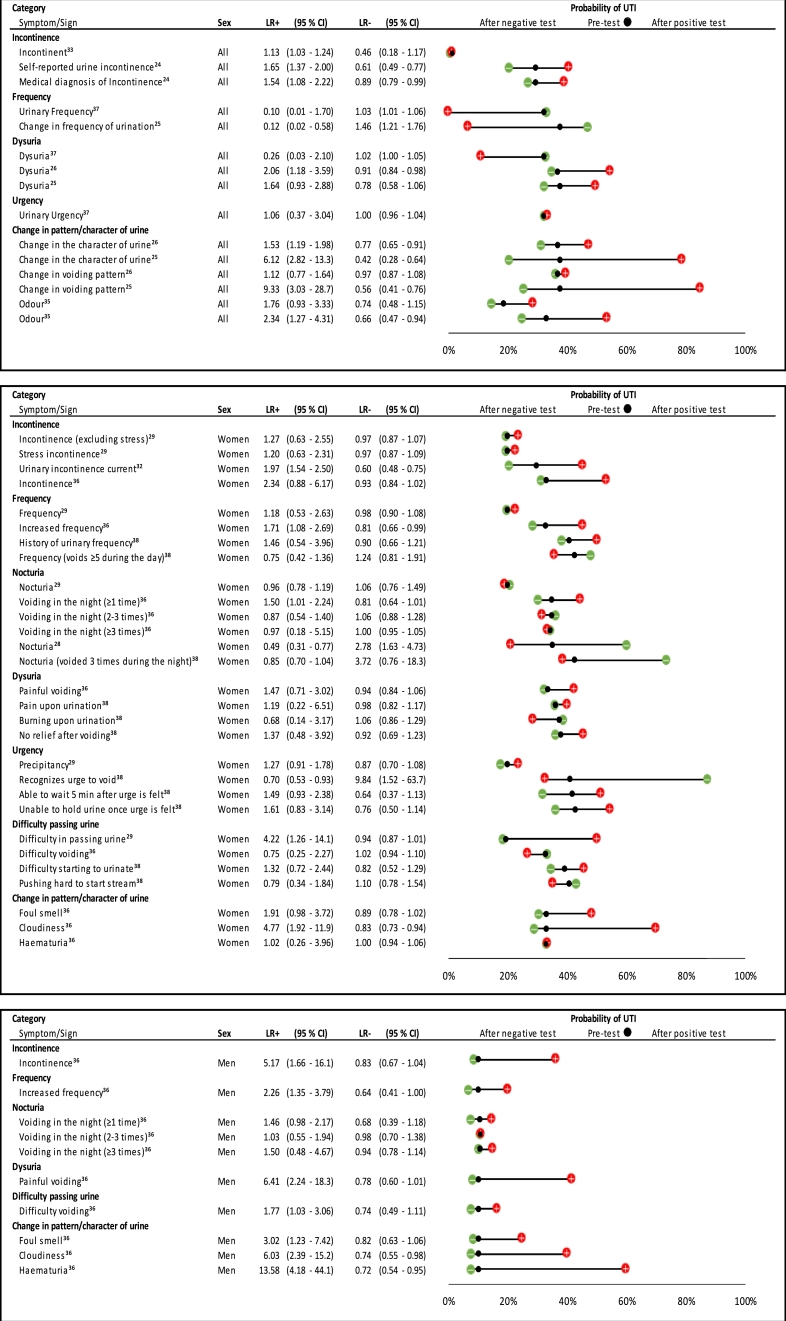
Likelihood ratios and pre- and post-test probabilities for urinary symptoms in predicting UTI). When possible, gender specific estimates have been presented; the dumbbell plots have been separated according to sex [Male and female combined (‘All’); women; and men]. Within each plot, symptoms have been divided into categories. Positive and negative likelihood ratios with 95% confidence intervals are presented for each symptom. The black dot within the dumbbell plot represents the pre-test probability of UTI (i.e. prevalence). The red dot represents the probability of UTI after a positive test (i.e. given that the symptom is present), and the green dot represents the probability of UTI after a negative test (i.e. given that the symptom is absent).

**Fig. 4:**
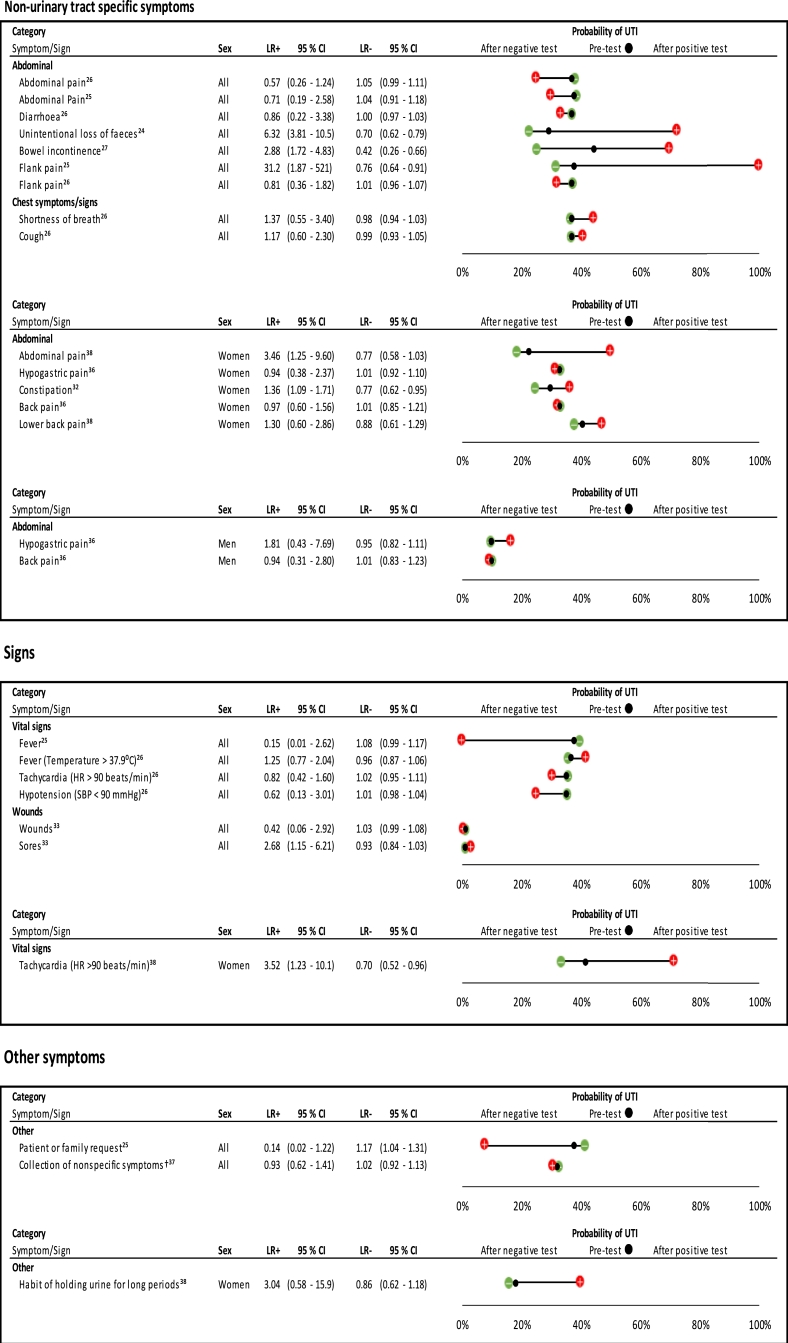
Likelihood ratios and probability plot for non-urinary tract symptoms and signs in predicting UTI. Plots have been separated according to symptom/sign category, and then ordered according to gender within each category. Likelihood ratios with 95% confidence intervals, and pre- and post-test probability of UTI given presence or absence of a symptom/sign, are presented. † Non-specific symptoms include: fatigue, restlessness, confusion, aggressiveness, loss of appetite, frequent falls, not being herself/himself HR: heart rate; SBP: systolic blood pressure.

**Fig. 5:**
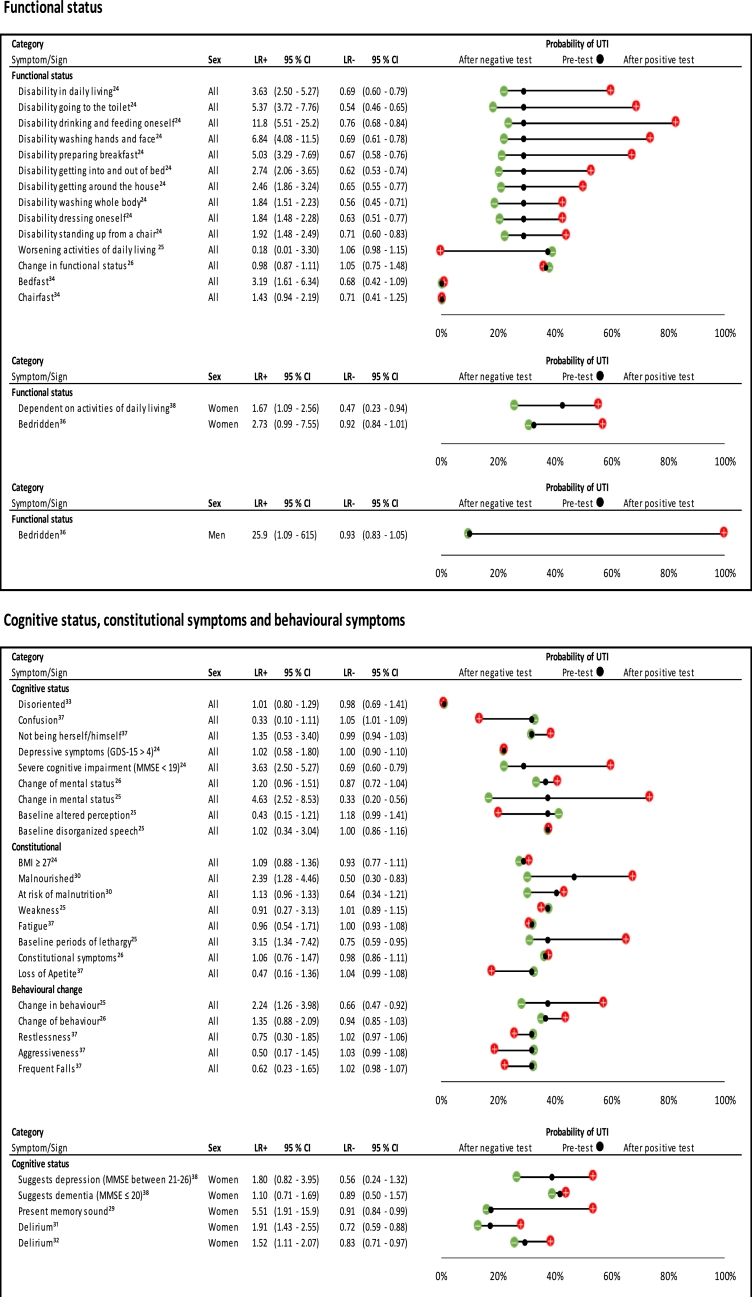
Likelihood ratios and probability plot for markers of functional and cognitive status in predicting UTI. Plots have been separated according to symptom/sign category, and then ordered according to gender within each category. Likelihood ratios with 95% confidence intervals, and pre- and post-test probability of UTI given presence or absence of a symptom/sign, are presented. BMI: body mass index; GDS-15: geriatric depression scale; MMSE: mini mental state examination.

**Fig. 6:**
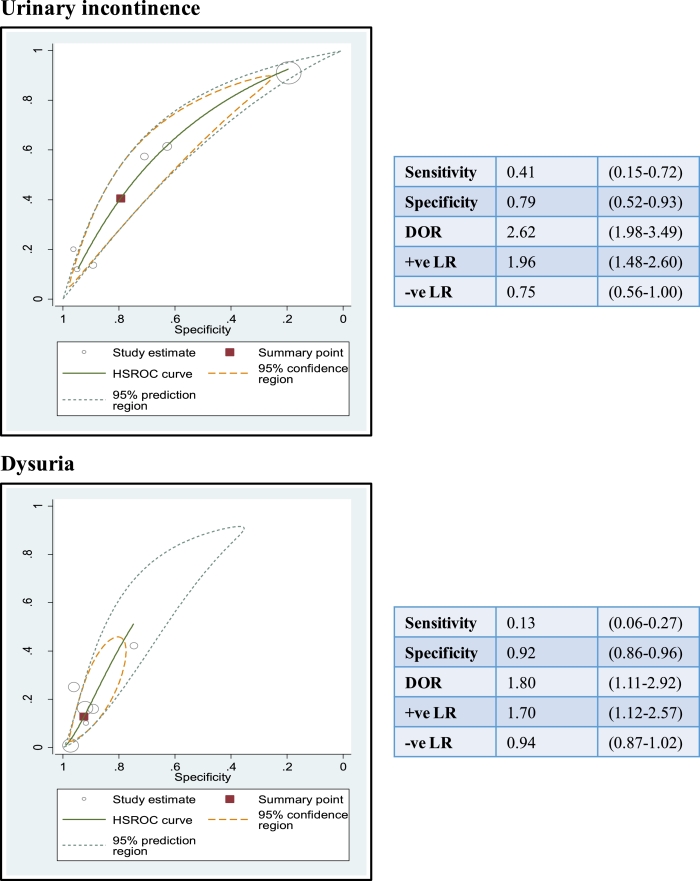
Summary receiver operating characteristic (ROC) curves for urinary incontinence and dysuria. ROC curves and summary statistics with 95% confidence intervals for urinary incontinence and dysuria in relation to UTI. Individual study estimates for both symptoms are represented by hollow circles. The summary point is represented by a red square. Summary statistics are presented within the boxes adjacent to the graphs DOR: diagnostic odds ratio; +’ve LR: positive likelihood ration; −’ve LR: negative likelihood ratio.

We identified 66 different symptoms and signs in relation to UTI (Figs. 3–5). There were sufficient studies to look at four symptoms using ROC plots (Fig. 6 and supplementary file 2). The other symptoms and signs have been presented as individual study estimates on dumbbell plots.

### Summary ROC plots

Both urinary incontinence and dysuria were predictors of UTI [+ ve LR: 1.96 (95% CI 1.48 – 2.60) and + ve LR: 1.70 (95% CI 1.12–2.57), respectively]. Absence of these symptoms did not help to rule out a diagnosis of UTI (Fig. 6). Fig. 6 shows that urinary incontinence and dysuria were quite specific, but not sensitive for UTI.

Summary ROC plots for both urinary frequency and nocturia revealed high heterogeneity and so we did not calculate pooled estimates for these symptoms (See supplementary file 2).

### Dumbbell plots

#### Urinary tract specific symptoms

Estimates for UTI specific symptoms were divided into seven broad categories, with gender specific estimates given when possible (Fig. 3). Much variation was found in the way that symptoms were described by studies. For example, incontinence was defined in six different ways, dysuria in five different ways, and urinary frequency was described in six different ways (Fig. 3).

Fig. 3 shows that when both sexes were assessed together, incontinence and a change in the character of urine were found to be predictors of UTI. One of the three estimates for dysuria produced a significant result.^26^ In women, of the four estimates for urinary incontinence, only one predicted UTI.^32^ This was also true of urinary frequency, where one of four estimates produced a significant result.^36^ Of the six estimates for nocturia in women, only one was significant in predicting UTI.^36^ None of the eight estimates for dysuria and urgency in women were significant,^29^, ^36^, ^38^ and absence of these symptoms did not help rule out UTI. Cloudy urine was a significant predictor of UTI in women, but not foul smelling urine or haematuria.

In men, there was only a single estimate for both urinary incontinence and frequency, but both produced significant results.^36^ Nocturia was not a predictor of UTI.^36^ However, unlike in women, dysuria was helpful in diagnosing UTI.^36^ Cloudy urine, foul smelling urine and haematuria were all predictors of UTI in men.^36^ Of note, all of the estimates in men came from a single study^36^ that was assessed as low risk against six of the seven QUADAS-2 domains (Fig. 2b).

#### Non-urinary tract specific symptoms

Fig. 4 shows that unintentional loss of faeces^24^ and bowel incontinence^27^ were predictors of UTI in all participants; however, diarrhoea^26^ or abdominal pain^25^^,^^26^ did not predict UTI. Fig. 4 also shows that in one study,^25^ flank pain was a large predictor of UTI (+ ve LR: 31.2 (95% CI 1.87–521) , but was not a predictor in another study.^26^ In women, abdominal pain^38^ and constipation^32^ were predictors of UTI, but back pain and hypogastric pain were not.^36^ Absence of bowel incontinence and unintentional loss of faeces in all participants helped to rule out UTI.^24^^,^^27^ Only one study assessed symptoms in males.^27^

#### Signs

Fig. 4 also shows that traditional signs associated with UTI (fever, tachycardia, and hypotension) were not predictors of UTI. In one study of 101 people, having a fever reduced the probability of UTI by 38 %.^25^ In women, a single study reported that tachycardia was significant.^38^

#### Markers of functional status

Fig. 5 shows that disability in performing a number of acts of daily living was a predictor of UTI in all participants; for example, estimates for disability in feeding oneself, disability in washing hands and face, disability in going to the toilet and disability in preparing breakfast were all significant. However, ten estimates of markers of functional status came from the same study, which had a moderate risk of bias.^24^ This study was conducted in the home setting and long-term care facilities. Being bedfast, but not chairfast, was found to predict UTI.^34^

Although being bedridden seems to be a predictor of UTI in men, the result is limited by the fact that only one participant in the study had the symptom in question, resulting in very wide confidence intervals (see Supplementary Table 3).^36^ Being bedridden was not a significant predictor of UTI in women.^36^

#### Cognitive status, behavioural symptoms and other symptoms

Fig. 5 shows that markers of cognitive status had limited use in predicting UTI in all participants, with two of the nine estimates producing significant results.^24^, ^25^ Only one of five estimates for a change in behaviour produced a significant result^25^. In women, delirium was a predictor of UTI, and absence of this symptom helped to rule out UTI.^31^, ^32^ Patient or family request to check for UTI did not help predict UTI and reduced the probability of UTI by 30% (Fig. 4).

## Discussion

### Main findings

The evidence from studies of predictors of infection in older adult outpatients is sparse, of variable quality and demonstrates that a number of urinary symptoms commonly associated with UTI, such as nocturia and urgency, have limited use for diagnosing UTI. In men, incontinence, foul smelling urine and haematuria were predictors of UTI, but not in women. Importantly, abnormal vital signs (fever, tachycardia and hypotension) are of limited value in UTI diagnosis. Symptoms that may be less typically associated with UTI, such as inability to perform a number of acts of daily living (poor functional status), were strong predictors of UTI. It must be noted, however, that the majority of these estimates were derived from a single study^24^ and are likely to be highly correlated with each other.

Haematuria was found to be of diagnostic value in diagnosing UTI in men, but not in women. We postulate that this could be a result of increased chance of contamination of urine specimens with blood in women, making it less discriminatory for UTI in females. Up to 40% of post-menopausal women have atrophic vaginitis,^39^ which can result in microscopic haematuria on urine dipstick. Urinary incontinence was found to be a strong predictor of UTI in men but not in women. This may be due to incontinence being approximately twice as common in women compared to men,^40^ and therefore less specific for the presence of UTI in women. Similarly, it may be that abnormal vital signs (such as fever, tachycardia and hypotension) were not found to be predictive of UTI, as if combined with other symptoms (such as breathlessness), they might better predict a different infection (such as a chest infection). We were only able to identify a single estimate for the value of combinations of symptoms in diagnosing UTI.^37^

We obtained a number of conflicting results for estimates of the same symptom in relation to UTI. One point estimate for urinary incontinence in the summary ROC plot was a distinct outlier.^33^ Incontinence was described in six different ways by included studies; including “self-reported,” “medical diagnosis of incontinence” and “incontinence (excluding stress).” The way in which incontinence was defined by the outlying study, and the potential impact of this on the point estimate is unclear.

Daley et al.^25^ found flank pain to be a predictor of UTI, whilst Juthani-Mehta et al.^26^ did not. There was also discrepancy between these two studies in their estimates for change in the character of urine and change in the voiding pattern. The reason for the difference in results between the studies is uncertain. The studies had similar prevalence of infection, study setting, age of participants, exclusion criteria and reference standard.

### Comparison with existing guidelines

A decision aid within SIGN guideline 88 supplementary material,^12^ aims to guide clinicians in managing older patients with fever. The aid suggests that in the absence of symptoms indicative of respiratory, gastrointestinal, or skin or soft tissue infection, two or more of the following symptoms suggest that UTI is likely: dysuria, urgency, frequency, urinary incontinence, shaking/chills, flank/suprapubic pain, frank haematuria and new onset or worsening or pre-existing confusion or agitation. However, the findings from our review does not support the use of some of these symptoms. We did not find urgency to be predictive of UTI; nor did we find dysuria, frequency, incontinence or haematuria to predict UTI in women. However, only one of the studies in our review assessed the diagnostic use of combinations of symptoms in diagnosing infection.^37^ Furthermore, the symptoms assessed in this review were not in the context of fever. The SIGN guideline decision aid was developed by the Scottish Antimicrobial Prescribing Group.^41^ Of note, they cite five references that were used to formulate the guidance: two from the Health Protection Agency,^42^, ^43^ SIGN guideline 88,^12^ one qualitative case study,^44^ and one cluster randomised clinical trial.^45^

## Strengths and limitations

To our knowledge, this is the first systematic review and meta-analysis assessing the utility of symptoms and signs in diagnosing UTI in older adult outpatients. Our search strategy was broad, in order to maximise chances of capture of relevant studies. We contacted several authors of studies to clarify details in the papers being screened for inclusion. Dual extraction of all data was performed. Data was extracted as fully as possible, providing gender specific data when possible. However, due to the breadth of the review, and given the difficulty of search terms in this area it is highly likely we have missed studies, especially unpublished studies. Whilst we were able to extract data for a wide range of symptoms and signs, due to variations in the way they were reported, meta-analysis was only possible for two symptoms.

Our results should be interpreted with caution as a number of the included studies were of poor quality, there was high heterogeneity between the included studies, and few symptoms and signs were assessed across more than one study. We postulate that differences in study design, the low quality of a number of the included studies and differences in definition of symptoms and signs may explain the observed heterogeneity. Furthermore, we assessed multiple independent symptoms and signs; having applied 95% confidence intervals, we might expect that 1 in 20 of our estimates might produce significant results as a result of chance.

A number of the studies were rated as ‘high risk’ across a number of the QUADAS-2 domains. However, we also recognise that in studies assessing the diagnostic accuracy of symptoms and signs, by their very nature, it is difficult to entirely eliminate subjectivity and a degree of bias. For example, incorporation bias is largely unavoidable because diagnoses of infections are often made taking into account symptoms and signs, as well as test results. Partly as a result of this as well as lack of standardisation of methodology, the feasibility of pooling results from such studies is restricted by significant heterogeneity. Insufficient studies were included within the meta-analysis to allow for meaningful subgroup analysis to be performed to explore the high heterogeneity.

We also included some studies in which small numbers of participants were recorded as having cancer; these participants may have been immunosuppressed. However, we also recognise that a number of older people have malignancy as a co-morbidity. This might actually be more representative of real populations that clinicians treat.

We deliberately excluded studies conducted in A&E units in an attempt to make the results more relevant to primary care, and therefore may not be generalizable to emergency rooms. We included four studies that included a small proportion of the total participants under the age of 65 years. In one study,^33^ 11% of participants were under 65 years of age, however, the author informed us that the two by two table data remained essentially unchanged after excluding them. Given the author's assertions we can be confident that this would not have biased the estimates obtained. Another study^38^ had an age range of 64–97 years, and in another,^35^ participants ranged from 64 to 102 years. In the study by Sundvall et al.^37^ the age range was 63–100 years; the author was able to confirm that there was only one participant aged 63 years, and all others were aged 65 years and above. For one study,^27^ we were unable to contact the authors to clarify the age of participants in the paper, however, due to the setting being a nursing home, we were confident that the majority, if not all, participants would be over 65 years.

We extracted episodic data into two by two tables when we were unable to extract participant data for one study.^24^ This may have led to over-estimation of the number of people with UTI, as the number of episodes of occurrence of a symptom or sign did not equate to the number of participants who experienced the symptom or sign. This is unlikely to have affected sensitivity, specificity or likelihood ratios. It may, however, have led to over-estimation of the positive predictive value and under-estimation of the negative predictive value. Finally, we excluded non-English studies and may therefore have omitted studies otherwise suitable for inclusion.

## Implications for future research

In order to make robust conclusions about the diagnostic value of symptoms and signs in diagnosing UTI in older adults, additional high quality studies are required. More studies assessing the utility of combinations of different symptoms in diagnosing UTI would also be beneficial. It would be helpful if definitions of symptoms and signs were consistent across studies, or at least reported in full and transparently, to allow meaningful comparisons between studies and pooling of results. Building up the evidence base for the clinical predictors that predict UTI in older adult outpatients could facilitate robust guidance, generation of clinical prediction rules, and improve the accuracy of the diagnosis. Further studies in this area are needed in order to make confident assertions of the utility of different symptoms and signs in diagnosing these infections in older adults.

## Implications for clinical practice

Evidence from included studies suggests that a number of symptoms and signs traditionally thought to be associated with UTI, may be of limited diagnostic value in older adults. This review highlights the complexity of diagnosing UTI in older adults, and the breadth of their clinical presentations. The results of this review are based on evidence from studies of variable quality and therefore should be treated with caution. Further high quality studies, with large numbers of participants, need to be performed to corroborate these findings. This would assist clinicians with prompt and accurate diagnosis of true UTI, encouraging appropriate treatment with antimicrobials. Clarity in this area may also help clinicians to differentiate UTI from asymptomatic bacteriuria, reducing inappropriate antimicrobial prescriptions for the latter condition, thereby improving antimicrobial stewardship. It is unclear how application of information from this review might translate into improved diagnostic rates of UTI in older adults or improve appropriate antimicrobial prescribing.

## Conclusions

There is limited evidence of varying quality appraising the utility of a range of symptoms and signs in diagnosing UTI in older adult outpatients. A number of symptoms and signs traditionally associated with UTI such as urgency, nocturia and abnormal vital signs may be of limited diagnostic value in older adult outpatients. Less classical features, such as inability to perform a range of acts of daily living, might be better predictors of UTI. More evidence from high quality studies conducted in this area is needed.

## Appendix Supplementary materials

Supplementary files Image, application 1
