# Supplementary material for: Diagnostic value of symptoms and signs for identifying urinary tract infection in older adult outpatients: Systematic review and meta-analysis
Source: J Infect. 2018 Nov;77(5):379–90. doi: 10.1016/j.jinf.2018.06.012 (PMC6203890; doi:10.1016/j.jinf.2018.06.012)
Supplement: Supplementary file 1 — Supplementary files [file mmc1.zip › Supplementary_file_1_JOI.docx]

**Supplementary file 1 - Search strategy**

**Medline**

| **# ▲** | **Searches** | **Results** |
| --- | --- | --- |
| **1** | **exp Aged/** | **2506360** |
| **2** | **Geriatrics/** | **27765** |
| **3** | **Geriatric Assessment/** | **20184** |
| **4** | **(((old or older) adj2 (people or person? or patient? or adult?)) or ("old age" or elderly or geriatric* or senior? or veteran?)).ti,ab.** | **412536** |
| **5** | **exp Aged/** | **2506360** |
| **6** | **Geriatrics/** | **27765** |
| **7** | **Geriatric Assessment/** | **20184** |
| **8** | **(((old or older) adj2 (people or person? or patient? or adult?)) or ("old age" or elderly or geriatric* or senior? or veteran?)).ti,ab.** | **412536** |
| **9** | **5 or 6 or 7 or 8** | **2682522** |
| **10** | **Bacterial Infections/** | **63105** |
| **11** | **exp Sepsis/** | **100368** |
| **12** | **Soft Tissue Infections/** | **2672** |
| **13** | **exp Skin Diseases, Infectious/** | **115534** |
| **14** | **exp Staphylococcal Skin Infections/** | **4797** |
| **15** | **Pneumonia, Bacterial/** | **9393** |
| **16** | **Pneumonia/** | **40804** |
| **17** | **exp Respiratory Tract Infections/** | **315574** |
| **18** | **tuberculosis/ or tuberculosis, pulmonary/** | **140585** |
| **19** | **exp Intraabdominal Infections/** | **43825** |
| **20** | **exp Urinary Tract Infections/** | **40719** |
| **21** | **Pyelonephritis/** | **13522** |
| **22** | **exp Meningitis, Bacterial/** | **22369** |
| **23** | **endocarditis/ or exp endocarditis, bacterial/** | **25182** |
| **24** | **((bacterial or microbial) adj2 infection?).ti.** | **6316** |
| **25** | **bacter?emia.ti.** | **9442** |
| **26** | **(sepsis or septic shock or septic?emia).ti.** | **33437** |
| **27** | **systemic infection?.ti.** | **642** |
| **28** | **((serious or sever*) and infection?).ti.** | **6812** |
| **29** | **common infection?.ti.** | **182** |
| **30** | **((skin adj2 infection?) or cellulitis).ti.** | **4062** |
| **31** | **(((respiratory or chest) adj2 infection) or pneumonia or tuberculosis).ti.** | **160019** |
| **32** | **(((intraabdom* or intra-abdom* or abdom*) adj2 (infection? or abscess*)) or appendicitis or peritonitis or diverticulitis).ti.** | **27576** |
| **33** | **(urin* adj2 infection?).ti.** | **14207** |
| **34** | **(pyelonephritis or ((kidney or renal) adj2 infection?)).ti.** | **7837** |
| **35** | **meningitis.ti.** | **25217** |
| **36** | **endocarditis.ti.** | **18219** |
| **37** | **or/10-36** | **821208** |
| **38** | **"signs and symptoms"/ or lower urinary tract symptoms/** | **1572** |
| **39** | **neurobehavioral manifestations/ or confusion/ or delirium/** | **10645** |
| **40** | **Cough/** | **13164** |
| **41** | **exp Dyspnea/** | **16968** |
| **42** | **Tachypnea/** | **94** |
| **43** | **Respiratory Rate/** | **1559** |
| **44** | **exp Fever/** | **37402** |
| **45** | **Body Temperature/ or Hypothermia/ or Sweating/** | **59239** |
| **46** | **Dehydration/** | **11340** |
| **47** | **Abdominal Pain/** | **16053** |
| **48** | **Diarrhea/** | **41294** |
| **49** | **Constipation/** | **11432** |
| **50** | **Fecal Incontinence/** | **8351** |
| **51** | **Headache/** | **24027** |
| **52** | **erythema/ or exanthema/** | **15577** |
| **53** | **neurologic manifestations/ or meningism/** | **7601** |
| **54** | **nausea/ or vomiting/** | **26276** |
| **55** | **lower urinary tract symptoms/ or dysuria/ or urinary incontinence/** | **20571** |
| **56** | **Urinary Retention/** | **3787** |
| **57** | **Chest Pain/** | **10462** |
| **58** | **exp Weight Loss/** | **31720** |
| **59** | **Anorexia/** | **4399** |
| **60** | **Appetite/** | **6446** |
| **61** | **Edema/** | **35709** |
| **62** | **exp "Severity of Illness Index"/** | **186920** |
| **63** | **(sign? or symptom* or feature? or manifestation?).ti.** | **260276** |
| **64** | **"signs and symptoms".ti,ab.** | **32835** |
| **65** | **vital sign?.ti,ab.** | **9268** |
| **66** | **((clinical or specific or nonspecific or non-specific or typical or atypical or distinguish* or local* or sever*) adj3 (sign? or symptom* or feature? or manifestation?)).ti,ab.** | **379774** |
| **67** | **((infection? or infectious? or bacter?emia or sepsis or septic*) adj5 (sign? or symptom* or feature? or manifestation?)).ti,ab.** | **37884** |
| **68** | **((cardiac or cardiovascular or neurolog* or respirator* or urin*) adj5 (sign? or symptom* or feature? or manifestation?)).ti,ab.** | **89906** |
| **69** | **(severity adj3 (illness or infection?)).ti,ab.** | **13823** |
| **70** | **(confusion or confused or delirium or delirious).ti,ab.** | **44324** |
| **71** | **(cough or tachypne* or tachypnoe* or dyspne* or dyspnoe* or respiratory rate or respiration rate or ((fast* or short* or difficult* or rate) adj2 breath*)).ti,ab.** | **84738** |
| **72** | **(tachycardi* or heart rate).ti,ab.** | **169341** |
| **73** | **(fever or febrile or pyrexia or pyrexic or body temperature).ti,ab.** | **173870** |
| **74** | **dehydrat*.ti,ab.** | **33562** |
| **75** | **capillary refill.ti,ab.** | **322** |
| **76** | **(abdominal pain or diarrhoea or diarrhea or vomit* or nausea or constipat* or ((faecal or fecal) adj incontinen*)).ti,ab.** | **184354** |
| **77** | **headache?.ti,ab.** | **64591** |
| **78** | **(rash* or erythema or exanthema).ti,ab.** | **46873** |
| **79** | **(meningism or ((stiff* or rigid*) adj2 (neck or nuchal))).ti,ab.** | **1742** |
| **80** | **(urin* adj (frequen* or retention or retain* or incontinen)).ti,ab.** | **8788** |
| **81** | **(ankle adj (edema* or oedema* or swell* or swollen)).ti,ab.** | **580** |
| **82** | **(edema or oedema).ti,ab.** | **107687** |
| **83** | **((weight adj (loss or lose)) or anorexia).ti,ab.** | **82468** |
| **84** | **((low* or decrease* or reduce*) adj2 (appetite or eat*)).ti,ab.** | **3117** |
| **85** | **chest pain?.ti,ab.** | **25843** |
| **86** | **((predictive or prognostic) adj factor?).ti,ab.** | **83904** |
| **87** | **((clinical or diagnos) adj2 (algorithm? or rule? or checklist? or check list? or tool? or score* or criteria)).ti,ab.** | **35003** |
| **88** | **((predict* or prognostic) adj2 (algorithm? or rule? or checklist? or check list? or tool? or score* or criteria)).ti,ab.** | **30077** |
| **89** | **((severity or severe) adj2 (algorithm? or rule? or checklist? or check list? or tool? or score* or criteria or index or assess* or classif*)).ti,ab.** | **36026** |
| **90** | **(risk adj (algorithm? or rule? or checklist? or check list? or tool? or score* or criteria or index)).ti,ab.** | **13765** |
| **91** | **(curb 65 or curb65 or crb 65 or crb65 or ((curb or crb) adj (scor* or criteria))).ti,ab.** | **397** |
| **92** | **(loeb adj2 (criteria or scor*)).ti,ab.** | **4** |
| **93** | **(medical early warning scor* or mews).ti,ab.** | **119** |
| **94** | **(pneumonia severity index or psi).ti,ab.** | **14182** |
| **95** | **or/38-94** | **1975464** |
| **96** | **Ambulatory Care/** | **37507** |
| **97** | **ambulatory care facilities/ or community health centers/** | **21334** |
| **98** | **general practice/ or family practice/** | **68186** |
| **99** | **Office Visits/** | **5803** |
| **100** | **Primary Health Care/** | **58709** |
| **101** | **general practitioners/ or physicians, family/ or physicians, primary care/** | **21425** |
| **102** | **(ambulatory adj3 (care or setting? or facilit* or ward? or department? or service?)).ti,ab.** | **13723** |
| **103** | **((general or family) adj2 (practi* or physician? or doctor?)).ti,ab.** | **98162** |
| **104** | **(primary care or primary health care or primary healthcare).ti,ab.** | **95104** |
| **105** | **(clinic? or office or visit? or health centre? or health center?).ti,ab.** | **411086** |
| **106** | **("out of hours" or ooh or "after hours").ti,ab.** | **3361** |
| **107** | **residential facilities/ or assisted living facilities/ or homes for the aged/ or exp nursing homes/** | **43139** |
| **108** | **Institutionalization/** | **4912** |
| **109** | **((nursing or residential or longterm or long-term or institutional) adj2 home?).ti,ab.** | **27256** |
| **110** | **((residential or longterm or long-term) adj (care or facilit*)).ti,ab.** | **18687** |
| **111** | **(care home? or home care or homecare).ti,ab.** | **17465** |
| **112** | **exp Home Care Services/** | **41593** |
| **113** | **((home or domiciliary) adj (visit* or call*)).ti,ab.** | **6379** |
| **114** | **(presenting or presentation).ti.** | **91304** |
| **115** | **((clinical or diagnos* or feature?) adj2 (presenting or presentation)).ti,ab.** | **58166** |
| **116** | **or/96-115** | **873442** |
| **117** | **37 and 95 and 116** | **12393** |
| **118** | **Community-Acquired Infections/** | **11198** |
| **119** | **((community acquired or home acquired) adj2 (infection? or pneumonia)).ti,ab.** | **9230** |
| **120** | **118 or 119** | **14976** |
| **121** | **120 and 95** | **4214** |
| **122** | **117 or 121** | **15962** |
| **123** | **9 and 122** | **5405** |

**Embase**

| **# ▲** | **Searches** | **Results** |
| --- | --- | --- |
| **1** | **exp aged/** | **2372943** |
| **2** | **geriatrics/** | **39459** |
| **3** | **geriatric assessment/ or geriatric care/** | **23390** |
| **4** | **(((old or older) adj2 (people or person? or patient? or adult?)) or ("old age" or elderly or geriatric* or senior? or veteran?)).ti,ab.** | **561942** |
| **5** | **1 or 2 or 3 or 4** | **2645534** |
| **6** | ***bacterial infection/** | **57283** |
| **7** | **exp sepsis/** | **194382** |
| **8** | ***soft tissue infection/** | **2772** |
| **9** | ***skin infection/ or exp *bacterial skin disease/ or *cellulitis/** | **52490** |
| **10** | ***pneumonia/** | **42471** |
| **11** | ***bacterial pneumonia/** | **4895** |
| **12** | **exp *respiratory tract infection/** | **210147** |
| **13** | ***tuberculosis/ or *lung tuberculosis/** | **125693** |
| **14** | **exp *abdominal infection/** | **6974** |
| **15** | ***appendicitis/ or *acute appendicitis/** | **14245** |
| **16** | **exp *urinary tract infection/** | **38622** |
| **17** | **exp *pyelonephritis/** | **12498** |
| **18** | ***endocarditis/ or *bacterial endocarditis/** | **23849** |
| **19** | **((bacterial or microbial) adj2 infection?).ti.** | **7859** |
| **20** | **bacter?emia.ti.** | **10980** |
| **21** | **(sepsis or septic shock or septic?emia).ti.** | **43904** |
| **22** | **systemic infection?.ti.** | **736** |
| **23** | **((serious or sever*) and infection?).ti.** | **8762** |
| **24** | **common infection?.ti.** | **216** |
| **25** | **((skin adj2 infection?) or cellulitis).ti.** | **5088** |
| **26** | **(((respiratory or chest) adj2 infection) or pneumonia or tuberculosis).ti.** | **175438** |
| **27** | **(((intraabdom* or intra-abdom* or abdom*) adj2 (infection? or abscess*)) or appendicitis or peritonitis or diverticulitis).ti.** | **30850** |
| **28** | **(urin* adj2 infection?).ti.** | **20046** |
| **29** | **(pyelonephritis or ((kidney or renal) adj2 infection?)).ti.** | **9033** |
| **30** | **meningitis.ti.** | **27156** |
| **31** | **endocarditis.ti.** | **20798** |
| **32** | **or/6-31** | **784296** |
| **33** | **exp symptom/ or symptom assessment/** | **101199** |
| **34** | **exp confusion/ or exp delirium/ or consciousness disorder/** | **50983** |
| **35** | **coughing/** | **72186** |
| **36** | **breathing disorder/ or exp dyspnea/ or exp tachypnea/** | **123473** |
| **37** | **breathing rate/** | **24065** |
| **38** | **body temperature disorder/ or fever/** | **167547** |
| **39** | **dehydration/** | **30739** |
| **40** | **exp abdominal pain/** | **113266** |
| **41** | **exp diarrhea/** | **195079** |
| **42** | **exp "nausea and vomiting"/** | **257550** |
| **43** | **Constipation/** | **66138** |
| **44** | **feces incontinence/** | **15810** |
| **45** | **Headache/** | **159698** |
| **46** | **exp erythema/ or rash/** | **143181** |
| **47** | **meningism/** | **1053** |
| **48** | **lower urinary tract symptom/** | **8528** |
| **49** | **exp urine incontinence/** | **59448** |
| **50** | **dysuria/ or urine retention/** | **27939** |
| **51** | **thorax pain/** | **59567** |
| **52** | **exp weight reduction/** | **121295** |
| **53** | **Anorexia/** | **47554** |
| **54** | **edema/ or ankle edema/** | **79143** |
| **55** | **disease severity/ or general condition deterioration/** | **417659** |
| **56** | **scoring system/** | **195377** |
| **57** | **(sign? or symptom? or feature? or manifestation?).ti.** | **293779** |
| **58** | **"signs and symptoms".ti,ab.** | **44314** |
| **59** | **vital sign?.ti,ab.** | **16922** |
| **60** | **((clinical or specific or nonspecific or non-specific or typical or atypical or distinguish* or local* or sever*) adj3 (sign? or symptom* or feature? or manifestation?)).ti,ab.** | **518387** |
| **61** | **((infection? or infectious? or bacter?emia or sepsis or septic*) adj5 (sign? or symptom* or feature? or manifestation?)).ti,ab.** | **48903** |
| **62** | **((cardiac or cardiovascular or neurolog* or respirator* or urin*) adj5 (sign? or symptom* or feature? or manifestation?)).ti,ab.** | **128098** |
| **63** | **(severity adj3 (illness or infection?)).ti,ab.** | **19025** |
| **64** | **(confusion or confused or delirium or delirious).ti,ab.** | **61181** |
| **65** | **(cough or tachypne* or tachypnoe* or dyspne* or dyspnoe* or respiratory rate or respiration rate or ((fast* or short* or difficult* or rate) adj2 breath*)).ti,ab.** | **130653** |
| **66** | **(tachycardi* or heart rate).ti,ab.** | **225983** |
| **67** | **(fever or febrile or pyrexia or pyrexic or body temperature).ti,ab.** | **229678** |
| **68** | **dehydrat*.ti,ab.** | **40921** |
| **69** | **capillary refill.ti,ab.** | **498** |
| **70** | **(abdominal pain or diarrhoea or diarrhea or vomit* or nausea or constipat* or ((faecal or fecal) adj incontinen*)).ti,ab.** | **268784** |
| **71** | **headache?.ti,ab.** | **96717** |
| **72** | **(rash* or erythema or exanthema).ti,ab.** | **71829** |
| **73** | **(meningism or ((stiff* or rigid*) adj (neck or nuchal))).ti,ab.** | **773** |
| **74** | **(urin* adj (symptom? or sign? or frequen* or retention or retain* or incontinen)).ti,ab.** | **18601** |
| **75** | **(ankle adj (edema* or oedema* or swell* or swollen)).ti,ab.** | **854** |
| **76** | **(edema or oedema).ti,ab.** | **148006** |
| **77** | **((weight adj (loss or lose)) or anorexia).ti,ab.** | **121254** |
| **78** | **((low* or decrease* or reduce*) adj2 (appetite or eat*)).ti,ab.** | **4997** |
| **79** | **chest pain?.ti,ab.** | **40726** |
| **80** | **((predictive or prognostic) adj factor?).ti,ab.** | **123983** |
| **81** | **((clinical or diagnos) adj2 (algorithm? or rule? or checklist? or check list? or tool? or score* or criteria)).ti,ab.** | **51607** |
| **82** | **((predict* or prognostic) adj2 (algorithm? or rule? or checklist? or check list? or tool? or score* or criteria)).ti,ab.** | **45484** |
| **83** | **((severity or severe) adj2 (algorithm? or rule? or checklist? or check list? or tool? or score* or criteria or index or assess* or classif*)).ti,ab.** | **54708** |
| **84** | **(risk adj (algorithm? or rule? or checklist? or check list? or tool? or score* or criteria or index)).ti,ab.** | **24381** |
| **85** | **(curb 65 or curb65 or crb 65 or crb65 or ((curb or crb) adj (scor* or criteria))).ti,ab.** | **853** |
| **86** | **(loeb adj2 (criteria or scor*)).ti,ab.** | **7** |
| **87** | **(medical early warning scor* or mews).ti,ab.** | **238** |
| **88** | **(pneumonia severity index or psi).ti,ab.** | **13388** |
| **89** | **or/33-88** | **3238943** |
| **90** | **ambulatory care/** | **32033** |
| **91** | **health care facility/ or health center/** | **82482** |
| **92** | **general practice/** | **73813** |
| **93** | **outpatient department/** | **47587** |
| **94** | **primary medical care/ or primary health care/** | **122426** |
| **95** | ***physician/ or general practitioner/** | **120515** |
| **96** | **general practitioners/ or physicians, family/ or physicians, primary care/** | **71958** |
| **97** | **(ambulatory adj3 (care or setting? or facilit* or ward? or department? or service?)).ti,ab.** | **17636** |
| **98** | **((general or family) adj2 (practi* or physician? or doctor?)).ti,ab.** | **124053** |
| **99** | **(primary care or primary health care or primary healthcare).ti,ab.** | **121270** |
| **100** | **(clinic? or office or visit? or health centre? or health center?).ti,ab.** | **603212** |
| **101** | **("out of hours" or ooh or "after hours").ti,ab.** | **4567** |
| **102** | **assisted living facility/ or nursing home/ or residential home/ or home for the aged/** | **56053** |
| **103** | **((nursing or residential or longterm or long-term or institutional) adj2 home?).ti,ab.** | **34284** |
| **104** | **((residential or longterm or long-term) adj (care or facilit*)).ti,ab.** | **23264** |
| **105** | **(care home? or home care or homecare).ti,ab.** | **20500** |
| **106** | **home care/** | **51189** |
| **107** | **((home or domiciliary) adj (visit* or call*)).ti,ab.** | **8086** |
| **108** | **(presenting or presentation).ti.** | **113991** |
| **109** | **((clinical or diagnos* or feature?) adj2 (presenting or presentation)).ti,ab.** | **81896** |
| **110** | **or/90-109** | **1291527** |
| **111** | **32 and 89 and 110** | **17805** |
| **112** | **community acquired infection/** | **874** |
| **113** | **community acquired pneumonia/** | **10188** |
| **114** | **((community acquired or home acquired) adj2 (infection? or pneumonia)).ti,ab.** | **13151** |
| **115** | **112 or 113 or 114** | **17376** |
| **116** | **89 and 115** | **7900** |
| **117** | **111 or 116** | **24621** |
| **118** | **5 and 117** | **6321** |

**WoK**

| **Set** | **Results** | **Save search history and/or create an alertOpen a saved search history** |
| --- | --- | --- |
| **1** | **255,204** | **TS=(((old or older) NEAR2 (people or person? or patient? or adult?)) or ("old age" or elderly or geriatric* or senior? or veteran?))** |
| **2** | **118,358** | **TS=(((bacterial or microbial) NEAR2 infection?)) OR TS=bacter?emia OR TS=(sepsis or septic shock or septic?emia) OR TS=(systemic infection?) OR TS=(((serious or sever*) NEAR2 infection?)) OR TI=(((serious or sever*) AND infection?))** |
| **3** | **305,685** | **TS=(((skin NEAR2 infection?) or cellulitis)) OR TS=((((respiratory or chest) NEAR2 infection) or pneumonia or tuberculosis)) OR TS=((((intraabdom* or intra-abdom* or abdom*) NEAR2 (infection? or abscess*)) or appendicitis or peritonitis or diverticulitis)) OR TS=((urin* NEAR2 infection?)) OR TS=((pyelonephritis or ((kidney or renal) NEAR2 infection?))) OR TS=(meningitis) OR TS=(endocarditis)** |
| **4** | **406,985** | **3 OR 2** |
| **5** | **251,061** | **TI=((sign? or symptom? or feature? or manifestation?)) OR TS=(((clinical or specific or nonspecific or non-specific or typical or atypical or distinguish* or local* or sever*) NEAR5 (sign? or symptom? or feature? or manifestation?))) OR TS=("vital sign*" OR "signs AND symtoms") OR TS=((infection? or infectious? or bacter?emia or sepsis or septic*) NEAR5 (sign? or symptom* or feature? or manifestation?)) OR TS=((cardiac or cardiovascular or neurolog* or respirator* or urin*) NEAR5 (sign? or symptom* or feature? or manifestation?))** |
| **6** | **628,126** | **TS=(confusion or confused or delirium or delirious) OR TS=((cough or tachypne* or tachypnoe* or dyspne* or dyspnoe* or ((fast* or short* or difficult*) NEAR2 breath*))) OR TS=(tachycardi*) OR TS=(fever or pyrexia or pyrexic) OR TS=(dehydrat* OR "capillary refill") OR TS=((abdominal pain or diarrhoea or diarrhea or vomit* or nausea or constipat* or ((faecal or fecal) NEAR incontinen*))) OR TS=((headache? or meningism or ((stiff* or rigid*) NEAR (neck or nuchal)))) OR TS=((rash* or erythema or exanthema)) OR TS=((urin* NEAR2 (symptom? or sign? or frequen* or retention or retain* or incontinen))) OR TS=(edema or oedema) OR TS=(((weight NEAR2 (loss or lose)) or anorexia OR ((lose or loss or reduc* or decreas*) NEAR2 appetite)))** |
| **7** | **158,529** | **TS=("predictive factor*" OR "prognostic factor*") or TS=(((clinical or diagnos) NEAR2 (algorithm? or rule? or checklist? or "check list?" or tool? or score* or criteria))) or TS=((predict* or prognostic) NEAR2 (algorithm? or rule? or checklist? or "check list?" or tool? or score* or criteria)) or TS=((severity or severe) NEAR2 (algorithm? or rule? or checklist? or "check list?" or tool? or score* or criteria or index or assess* or classif*)) or TS=(risk NEAR1 (algorithm? or rule? or checklist? or check list? or tool? score* or criteria or index)) or TS=(curb 65 or curb65 or crb 65 or crb65 or ((curb or crb) NEAR1 (scor* or criteria))) OR TS=(loeb NEAR2 (criteria or scor*)) OR TS=("medical early warning scor*" or mews or "pneumonia severity index" or psi)** |
| **8** | **1,017,184** | **7 OR 6 OR 5** |
| **9** | **1,488** | **8 AND 4 AND 1** |
