# Supplementary material for: Diagnostic value of symptoms and signs for identifying urinary tract infection in older adult outpatients: Systematic review and meta-analysis
Source: J Infect. 2018 Nov;77(5):379–90. doi: 10.1016/j.jinf.2018.06.012 (PMC6203890; doi:10.1016/j.jinf.2018.06.012)
Supplement: Supplementary file 1 — Supplementary files [file mmc1.zip › Supplementary_file_2_JOI.docx.pptx]

## Slide 1
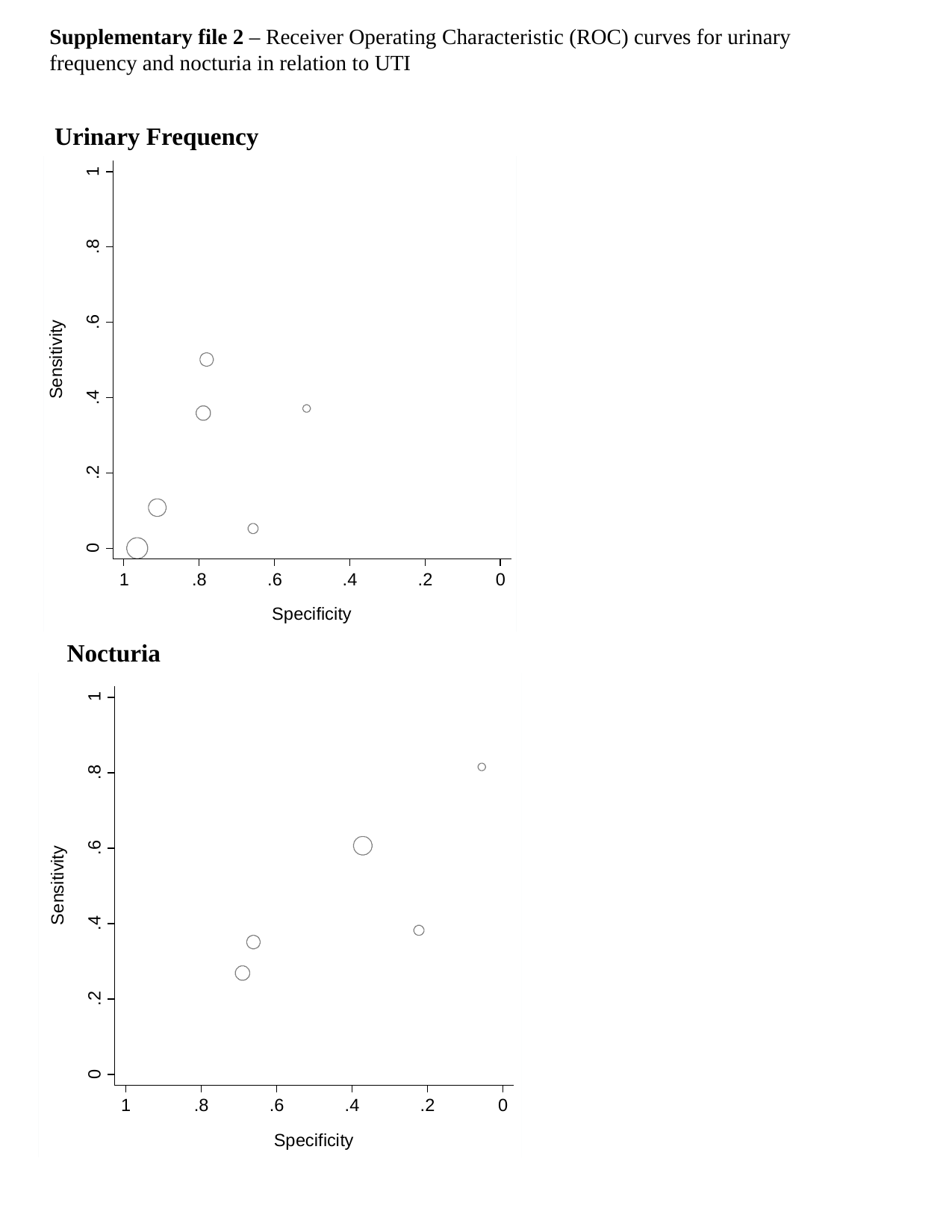

Supplementary file 2 – Receiver Operating Characteristic (ROC) curves for urinary frequency and nocturia in relation to UTI
Urinary Frequency
Nocturia
