# Supplementary figures and images for: Diagnostic value of symptoms and signs for identifying urinary tract infection in older adult outpatients: Systematic review and meta-analysis
Source: J Infect. 2018 Nov;77(5):379–90. doi: 10.1016/j.jinf.2018.06.012 (PMC6203890; doi:10.1016/j.jinf.2018.06.012)

## Slide 1
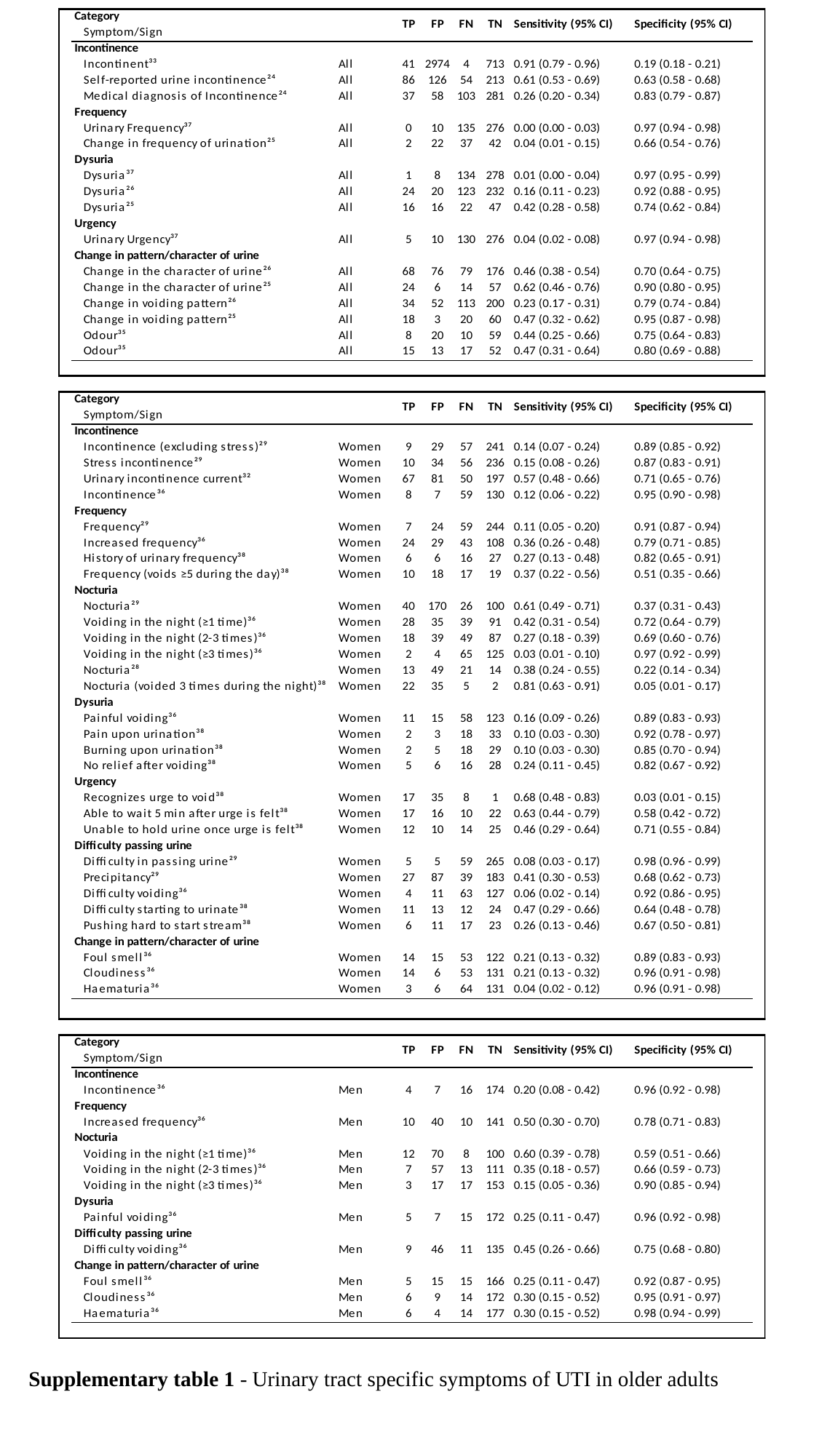

Supplementary table 1 - Urinary tract specific symptoms of UTI in older adults

Supplement: Supplementary file 1 — Supplementary files [file mmc1.zip › Supplementary_file_3_JOI.docx.pptx]

## Slide 1
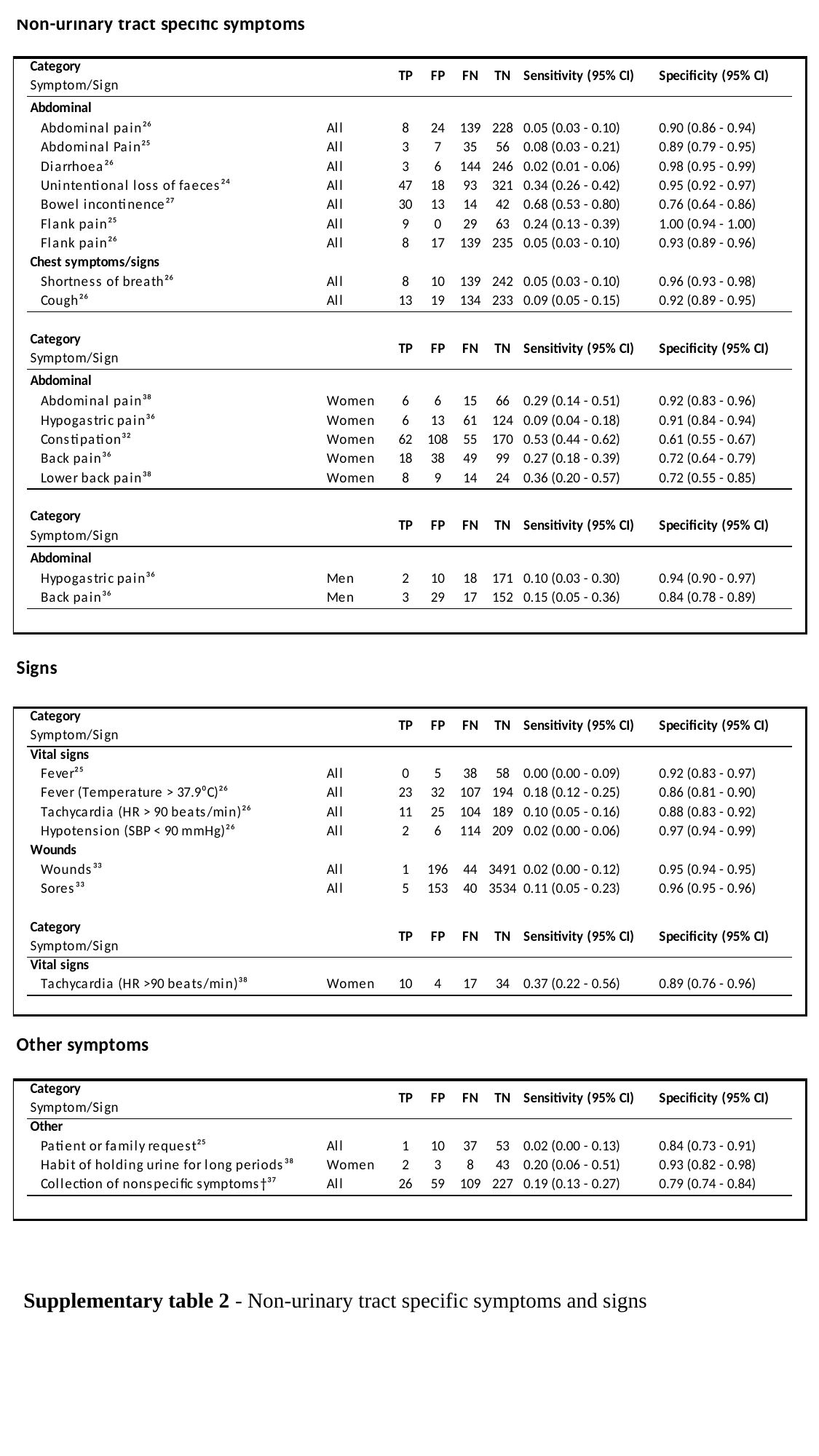

Supplementary table 2 - Non-urinary tract specific symptoms and signs

Supplement: Supplementary file 1 — Supplementary files [file mmc1.zip › Supplementary_file_4_JOI.docx.pptx]
